# Ambitions for palliative and end of life care: mapping examples of use of the framework across England

**DOI:** 10.1186/s12904-023-01207-3

**Published:** 2023-06-29

**Authors:** Erica Borgstrom, Joanne Jordan, Claire Henry

**Affiliations:** The Open University, Walton Hall, Milton Keynes, Buckinghamshire, MK7 6AA UK

**Keywords:** Ambitions Framework, Palliative care, End of life care, Examples of use, Policy, Service development and improvement, Questionnaires, Collaboration, Education and training, Mutual learning

## Abstract

**Background:**

Since 2015, the *Ambitions for Palliative and End of Life Care: a national framework for local action* has provided guidance for care within England and beyond. Relaunched in 2021, the Framework sets out six Ambitions which, collectively, provide a vision to improve how death, dying and bereavement are experienced and managed. However, to date, there has been no central evaluation of how the Framework and its Ambitions have been implemented within service development and provision. To address this evidence gap, we investigated understanding and use of the Framework.

**Methods:**

An online questionnaire survey was conducted to identify where the Framework has been used; examples of how it has been used; which Ambitions are being addressed; which foundations are being used; understanding of the utility of the Framework; and understanding of the opportunities and challenges involved in its use. The survey was open between 30 November 2021–31 January 2022, promoted via email, social media, professional newsletter and snowball sampling. Survey responses were analysed both descriptively, using frequency and cross-tabulations, and exploratively, using content and thematic analysis.

**Results:**

45 respondents submitted data; 86% were from England. Findings indicate that the Framework is particularly relevant to service commissioning and development across wider palliative and end of life care, with most respondents reporting a focus on Ambition 1 (Each person is seen as an individual) and Ambition 3 (Maximising comfort and wellbeing). Ambition 6 (Each community is prepared to help) was least likely to be prioritised, despite people welcoming the focus on community in national guidance. Of the Framework foundations, ‘Education and training’ was seen as most necessary to develop and/or sustain reported services. The provision of a shared language and collaborative work across sectors and partners were also deemed important. However, there is some indication that the Framework must give more prioritisation to carer and/or bereavement support, have greater scope to enhance shared practice and mutual learning, and be more easily accessible to non-NHS partners.

**Conclusions:**

The survey generated valuable summary level evidence on uptake of the Framework across England, offering important insights into current and past work, the factors impacting on this work and the implications for future development of the Framework. Our findings suggest considerable positive potential of the Framework to generate local action as intended, although difficulties remain concerning the mechanisms and resources necessary to enact this action. They also offer a valuable steer for research to further understand the issues raised, as well as scope for additional policy and implementation activity.

**Supplementary Information:**

The online version contains supplementary material available at 10.1186/s12904-023-01207-3.

## Background

### The ambitions framework

**Fig. 1:**
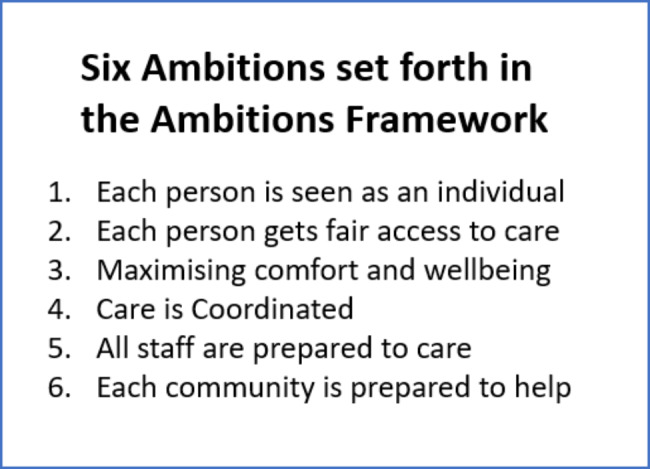
Six ambitions

The *Ambitions for Palliative and End of Life Care: a national framework for local action* provides guidance for care within England and beyond. It was first launched in 2015, co-produced by twenty-seven partners drawn from cross-sector health and social care [1]. It was relaunched in 2021 for an additional five years, with the partnership expanding to over thirty organisations. The Framework sets out six Ambitions (Fig. 1) which, collectively, provide a vision to improve how death, dying and bereavement are experienced and managed. The Ambitions are underpinned by a set of foundations, which offer guidance for action to be taken towards their realisation.

Figure adapted from an image provided by NHS England on behalf of the National Palliative and End of Life Care Partnership.

The Framework is the latest in the trajectory of palliative and end of life care policy in England [2–5]. Unlike the national *End of Life Care Strategy* [2], it is not mandatory. Instead, and in line with a localised approach, the Framework is supported by a self-assessment document [6], which can be used to self-assess provision against the Ambitions.

### How the ambitions framework has been used

A limited body of evidence addresses understanding and use of the Framework. A survey on impact of the Framework in England showed extensive use, especially for developing local strategies, although issues with implementation were identified [7]. Recommendations were made on how the Framework could be refined, but a consensus held that it should not be significantly changed to allow the continuation of relevant work [8].

The remaining literature addresses the Framework in three main ways. First, editorials [9, 10] and blogs [11–13] that summarise the aims and content of the Framework, typically highlighting its potential as a positive instrument for service change.

Second, examples of how the Framework has been used to enact service development and improvement [14–16] or to develop practice guidance [17]. These examples illustrate how explicit links between the Framework and action (either in service development or direct patient/public interaction) have been made. The majority of these examples of use are located in the grey literature, typically in the form of conference or workshop presentations. The current limited volume and diversity of this evidence base precludes a systematic review that might identify key issues concerning the impact of the Framework to date, or the implications for future development.

The third realm of literature provides a more critical analysis of how the Framework (loosely conceptualized as ‘policy’ or ‘guidance’ or ‘strategy’) can have meaningful impact. For example, interview-based research with professionals involved in the development of English policy in end of life care demonstrated broad support of the Framework in bringing together various strands of policy, identifying a common direction for work, and helping to progress service development at the local level including in respect of tackling inequalities in provision [18, 19]. However, several areas of concern were identified. These include the understandability of the Framework, its limitations as non-mandatory guidance and its place amongst other end of life and wider policy priorities, and how localism (in terms of service provision and policy settings) mitigates against consistent provision in the context of rising inequity [18, 19]. The evidence from this literature is consistent with that concerning both end of life care policy [6; 20; 21], as well as healthcare policy more generally [22]. The key questions raised throughout include how policy is implemented in practice, how one can understand or measure the impact of policy, and what (un)intended consequences there may be from the creation and use of policy.

### Our research

To date, there has been no central evaluation of how the Framework and its Ambitions have been implemented within service development and provision. To address this evidence gap, we investigated understanding and use of the Framework. Our objectives were to identify:

- where the Framework has been used (geographically and care settings);
- examples of how it has been used;
- which Ambitions are being addressed;
- which foundations are being used;
- understanding of the utility of the Framework;
- understanding of the opportunities and challenges involved in use of the Framework.

To address these objectives, we reviewed relevant literature and existing information about the Framework and undertook a national (England) online questionnaire survey (Appendix 1).

## Research design and methods

The survey collected data on understanding and use of the Framework and included a mapping of examples of relevant local activity. The questionnaire mainly consisted of closed questions, with some questions allowing for free text response. It utilised standardised descriptors (e.g., of geographical areas, care settings) in line with previous palliative and end of life care surveys administered by the National Programme for End of Life Care. Alongside the nature of the work undertaken, survey questions sought information on: primary Ambition(s) guiding any work; how the Framework was understood to enable this work; and, perceived challenges to use of the Framework. Questions were tested against a set of presentations from the Ambitions Partners monthly webinar series that highlights service examples.^1^ The questionnaire and encompassing research process was reviewed and pilot tested by our advisory group, which included members of the public, academics, and health and social care professionals.^2^

The survey was open between 30th November 2021 and 31st January 2022, hosted on the JISC Online Survey platform. Open and closing dates of the survey were influenced by ethics approval agreements and project funding period (project dates: start of October 2021 to end of March 2022). Invitations to participate were circulated multiple times electronically, using email and social media for snowball dissemination. The survey link was shared with the Ambition Partnership who were encouraged to share it amongst their networks. The invite was also included in the NHS England National Palliative and End of Life Care Update newsletter of January 2022.^3^ There was no target response rate nor targeted sampling.

To minimise missing data and facilitate withdrawal of data, the platform collected data only from respondents who completed the survey (i.e., clicked ‘submit’); they did not have to answer every question to submit their responses. Participants were informed that by clicking ‘submit’ they were consenting to their data being collected and that anonymised data would be used for publication; they had the option to opt-in to receive a copy of the project report and future research. Participant ID codes were autogenerated by the survey platform and do not contain any identifying information about the respondent.

To expedite survey completion, questions focused on core details of relevant work. Closed question data were analysed descriptively, using frequency and cross-tabulations within JISC Online. Open questions on policy context were coded by content to produce a quantitative overview. Other qualitative free-text data were analysed using thematic analysis [23] to capture core patterns in issues arising and concomitant understandings. All analyses were led by one author (E.B.), with input from the research team (C.H. & J.J.) to promote analytical rigour and the full possibilities for data interpretation [24].

## Findings

### Characteristics of ambitions related work overall

**Fig. 2:**
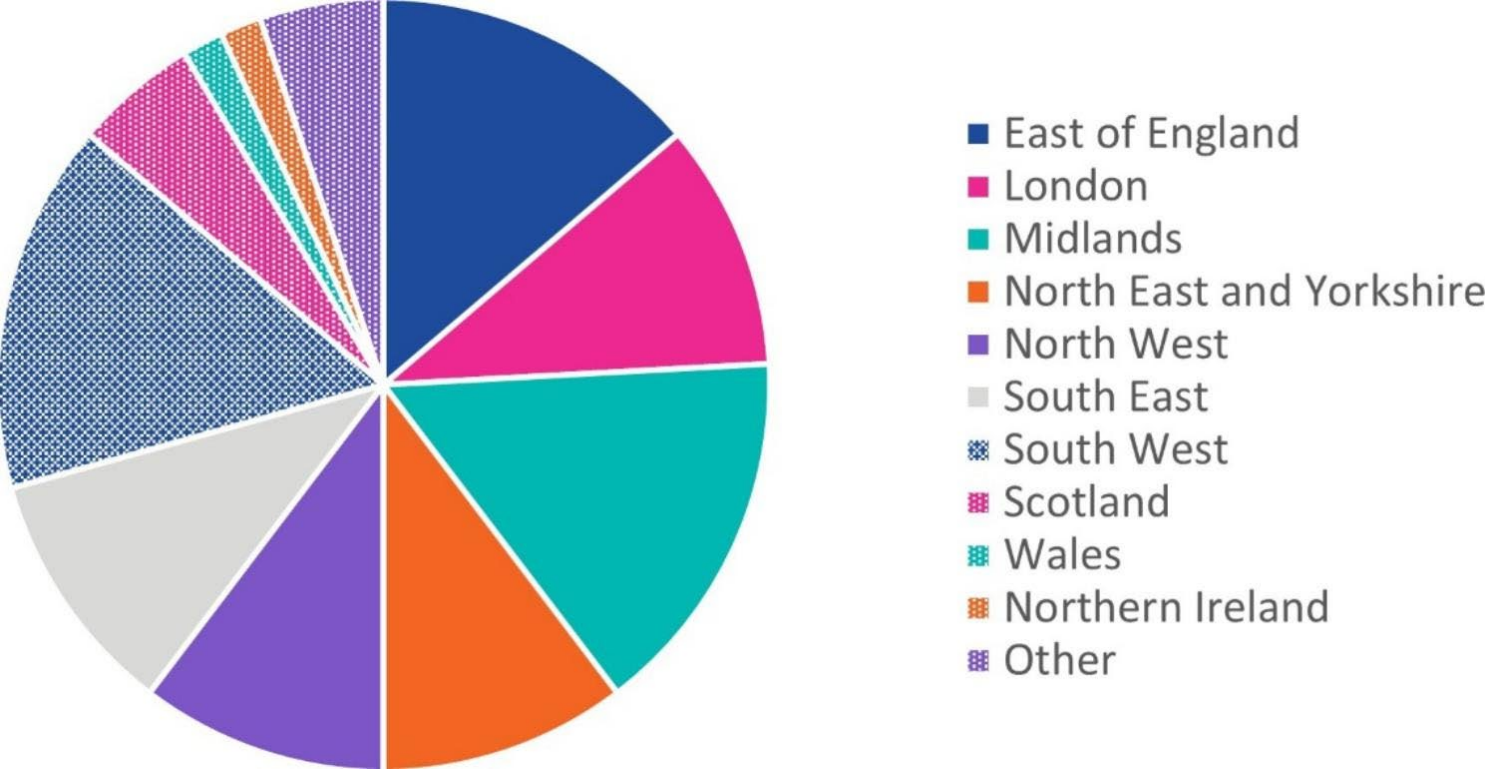
Location of Services

We received 45 responses. Over 86% of responses described services geographically distributed across England; for each region, between 6 and 9 examples of services were returned. Other areas of the UK were also represented, with examples received from Wales (n = 1), Northern Ireland (n = 1), Scotland (n = 3), and the Isle of Man (n = 1; provided in Other) (Fig. 2); respondents could select more than 1 location. Several respondents noted that their service provided national coverage, which could be only England or UK-wide; these are captured under ‘Other’. Most services were either NHS (73%) and/or charitably (57%) funded.

**Fig. 3:**
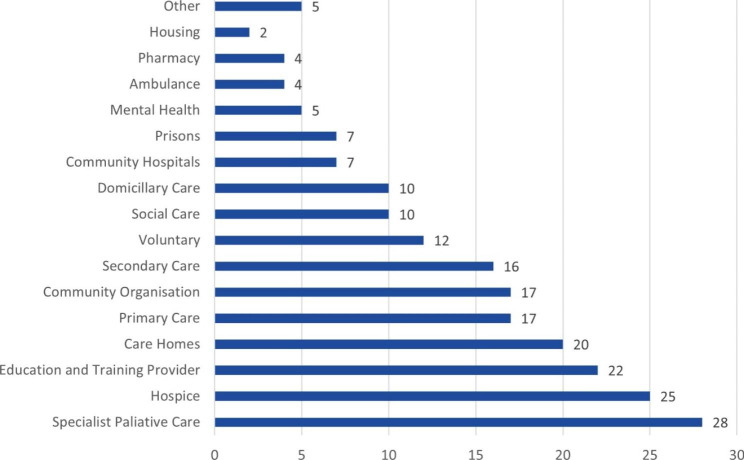
Service Setting* *More than one setting could be selected

Respondents were invited to identify the setting to which their service example related, with the option of selecting more than one setting (Fig. 3). We received examples of work from all settings listed although the majority of examples came from specialist palliative care and/or hospice care. Where respondents selected more than one setting, it is evident that work undertaken in settings outside of specialist services was supported by specialist palliative care. Approximately half of the examples indicated that multiple organisations were involved, and over 70% of respondents indicated wider stakeholder involvement (for example, patients, services users, clients and/or the public) when it came to service design.

**Fig. 4:**
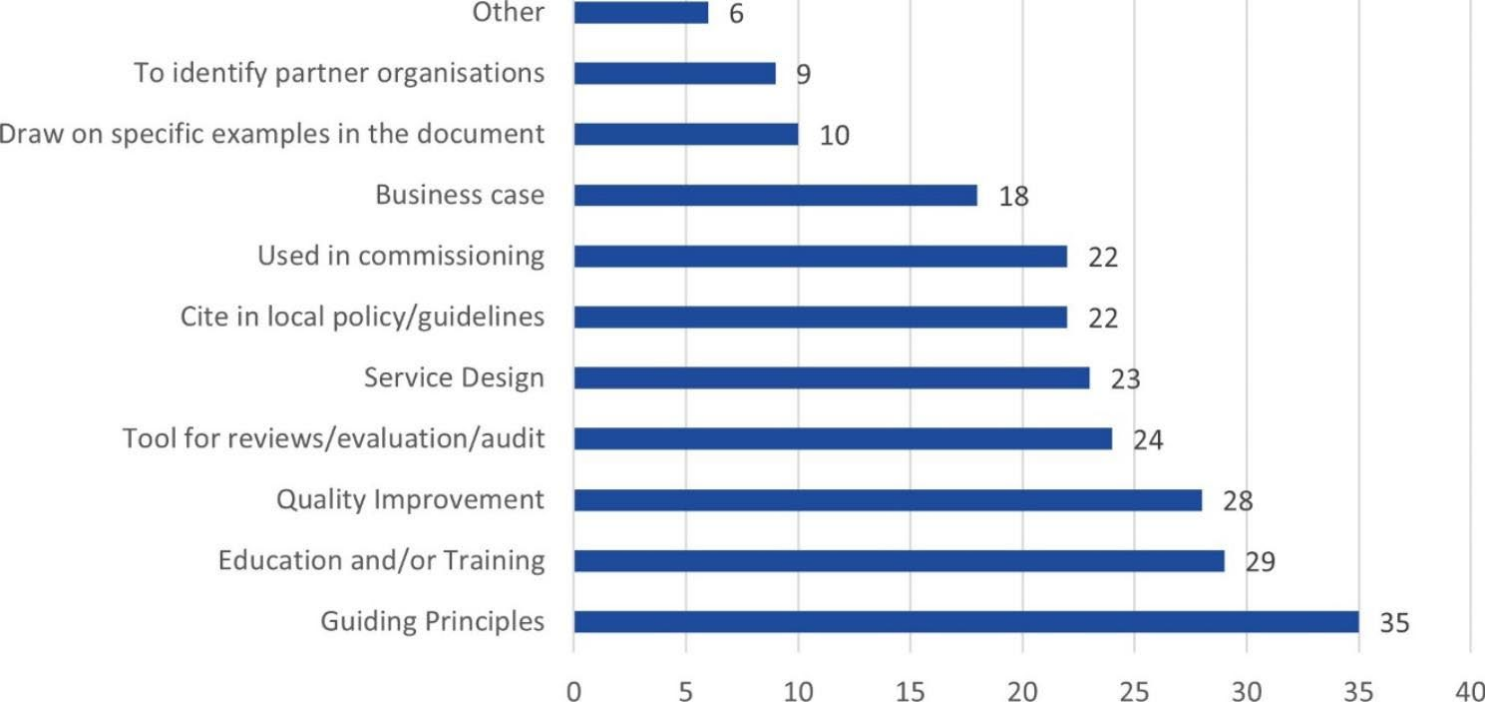
Service Setting* * More than one use could be selected

In terms of how the Framework was used to support service design and/or provision (Fig. 4), a majority of respondents reported its provision of guiding principles for the work undertaken. Use in supporting education and training, and in quality improvement, was also regularly highlighted. In addition, in a significant number of examples, the Framework was used in local policy development, service commissioning, and/or business case development. Most services (71%) were established before 2015, predating the first launch of the Framework.

Respondents were provided with a list of factors and asked to identify those needed to design and sustain the service examples reported. The listed factors were aligned to the eight foundations set out in the Framework: education and training, personalised care planning, leadership, evidence and information, shared records, those important to the dying person, 24/7 provision, and co-design. Education and training were noted as important by over 93% of respondents, with co-design being least frequently cited ( just over half of respondents). Overall, more than six foundations were identified as being useful for service design and sustaining services by all respondents. Respondents were able to identify ‘other’ aspects required; resources and carers were listed in free-text boxes.

To understand use of the Framework in a wider policy context, respondents were asked about other policy, guidance and frameworks supporting the design and/or delivery of services. Of the 34 responses provided to this question, most frequently cited was National Institute for Health and Care Excellence (NICE) guidance, followed by One Chance to Get it Right (also identified as Five Priorities [3]) and Care Quality Commission (CQC) frameworks and reports. Several other end of life care reports, as well as the national End of Life Care Strategy [2], were mentioned, with respondents noting their ongoing use. The ‘other’ category included 18 different items; each mentioned a single time. These included, but are not limited to: the Compassionate City/Civic Charter [25]), The Lantern Model of Care [26], individual pieces of research or service user feedback, and AgeUK quality specifications.

### Characteristics of use of each ambition

Ambition 1 was most frequently identified as the primary ambition guiding relevant work (n = 24/45).

Ambition 3 was the second most frequently cited primary ambition (n = 12/45). Ambitions 2 and 4 were both identified as the primary ambition in three cases. Ambition 5 was identified as the primary Ambition in two cases, and Ambition 6 in one case. Table 1 sets out the top three/four characteristics of different aspects of use for each of the six Ambitions.^4^

**Table 1:** Characteristics of use of the Ambitions

	How used	Settings of use	Factors (Foundations) necessary to support work	Combination with other Ambitions
Ambition 1 (Each person is seen as an individual)	(1) Provision of guiding principles (2) Quality improvement (3) Education & training	(1) Specialist palliative care (2) Education & training (3) Hospice	(1) Personalised care planning (2) Education & training (3) Those important to the dying person / Evidence & information	(1) Ambition 3 All other Ambitions also represented
Ambition 2 (Each person gets fair access to care)	(1) Provision of guiding principles (2) Quality improvement (3) A tool for review / Education & training	(1) Hospice All other settings (primary care, secondary care, care homes, specialist palliative care, domically care, education and training & community organisation) equally represented i.e. Ambition 2 being used across or with different settings and partners	(1) Personalised care planning (2) Shared records (3) Education & training (4) Co-design	All Ambitions equally represented
Ambition 3 (Maximising comfort and wellbeing)	(1) Provision of guiding principles (2) Quality improvement (3) Service design	(1) Specialist palliative care (2) Hospice (3) Care homes / Education & training / Community organisations	(1) Shared records (2) Education & training (3) Leadership	Ambitions 1, 2 & 4 considered more applicable than Ambitions 5 & 6 i.e., suggests services being organised around principles of person-centred care
Ambition 4 (Care is coordinated)	(1) A tool for review (2) Provision of guiding principles / Business case development / Identification of partner organisations / Education & training	(1) Primary care (2) Secondary care / Care homes / Social care / Specialist palliative care	(1) Evidence & information (2) Leadership (3) Personalised care planning / Those important to the dying person / Co-design	(1) Ambition 1 All other Ambitions also represented
Ambition 5 (All staff are prepared to care)	(1) Provision of guiding principles (2) Quality improvement (3) Education & training	(1) Secondary care (2) Education & training (3) Primary care/ Social care / Care homes / Hospice / Community hospitals / Specialist palliative care	(1) Education & training (2) Personalised care planning / Shared records / Evidence & information / Those important to the dying person / 24/7 care / Leadership	All Ambitions equally represented, except for Ambition 6 (not cited)
Ambition 6 (Each community is prepared to help)	(1) Provision of guiding principles / Cite in local policy / Identification of partner organisations / Education & training	All settings equally represented	(1) Personalised care planning / Evidence & information Those important to the dying person / Education & training / Co-design / Leadership	(1) Ambition 1 / Ambition 3

## Understandings of the ambitions framework

### Opportunities provided by the framework

Across all free-text responses (n = 41), understanding of how the Framework supported service development highlighted five main areas of benefit. First, its articulation and explicit endorsement of important end of life care values in the form of “guiding vision and principles” (Participant 729). These principles were considered central to the legitimisation of local level activity, all the more so as they were published under the auspices of a national body (NHS England). Second, its important contribution to raising awareness about the “need and importance” (Participant 149) of palliative and end of life care, especially for “raising the profile and complexity” (Participant 678) of its wide role and multiple levels and settings of provision. Third, its facilitation of “more focused and inclusive conversations” (Participant 858) by providing a “shared language” (Participant 149). This shared language was associated with enhanced communication with others working across the wider palliative and end of life care system, as illustrated in the following statement:

*“The Framework is a very useful guide we use when talking to commissioners / funders / the public about how care for people and their families can be improved.”* (Participant 934).

Enhanced communication was seen as fundamental to the development of local level collaboration. In the latter context, some respondents described bespoke local partnership groups, with the Framework being used to identify specific aims, outcomes, and partners.

The role of the Framework in enabling a process of service development from strategic conception to practical delivery was most frequently cited as an area of benefit. Several examples of this process were described, including use of the Framework to (re)develop local strategies, set the agenda for operational meetings, identify areas for service improvement, and secure additional funding for new or widening service provision. Some respondents noted their use of the Self-Assessment Tool to facilitate this process. Here, the Framework was described as a “benchmark” (Participant 371) and to provide a “baseline to measure progress against” (Participant 229). In this regard, several respondents highlighted the Framework’s role in consolidating the perceived value of service evaluation, with one claiming that it has “brought audit and review into everyday practice” (Participant 024) and another that “it has enabled me to give a very clear account as to what good looks like within the organization” (Participant 477).

The areas outlined above were not mutually exclusive. So that, for example, respondents could highlight the importance of the values legitimated through the Framework being useful for the development of partnership working, which was further facilitated by the Framework’s provision of a shared language through which service developments could be articulated. Moreover, whilst Ambitions 6 (about communities) was identified least frequently as a primary guiding ambition, in open text comments it was regularly endorsed, often being linked to the notion of compassionate communities.

### Challenges to use of the framework

Across all responses (n = 41), understanding of the challenges faced in use of the Framework are summarised in four main areas; people identified between 1 and 4 challenges in their response. First, a need for extensive promotion of the Framework to increase its use in training and other strategic service development and review contexts. To help with awareness raising and enhance the perceived relevance of the Framework, the creation of scaled down, more “user friendly” (Participant 678) versions was recommended. These were considered particularly important for individuals and organisations working outside of specialist palliative and end of life care. Other suggestions to promote awareness and use focused on the creation of opportunities for shared learning and dissemination of examples of relevant work. Several respondents suggested that more work could be done to use the Framework at both a national and local level to drive funding and other resources, including a national steer to Integrated Care Systems. To do so, one participant noted (697) the framework should be kept “in the forefront of any service developments”; others recommended revisiting it frequently in consultation with stakeholders to maintain its relevance.

Second, particularly in respect of Ambition 6 (Every community is prepared to help), a potential tension between the overall NHS England approach to palliative and end of life care and that of the compassionate communities movement, was highlighted. For example, when considering community work Participant 246 noted “the biggest challenge has been fitting into the restrictive boxes they [clinical commissioners in NHS] place on what is classed as ‘clinical benefit.‘” Participants noted an incongruence between how compassionate communities are envisioned and enacted and what is required within NHS ways of designing services. More broadly, the “daunting” prospect of using the Framework alongside other policy and regulation was noted. To help make connections and provide a clear steer, it was recommended that the Framework should be explicitly aligned with other guidance, such as NICE guidance and previous policy [3], and regulatory frameworks, such as the Care Quality Commission. In this context, it was acknowledged that the Framework supports the requirements of the National Audit of Care at the End of Life.

Third, a recurrent theme concerned the challenges associated with operationalisation of the Ambitions. For example,

*The foundations were easy and useful to operationalise and use as basis for service improvement and redesign. I have found the Ambitions more difficult to put into practice. Although I generally agree with the statements it is difficult to use them to design or measure services”* (Participant 230).

In terms of the content of the Framework document, relevant detail was considered lacking. Whilst some respondents considered the Framework to be helpful for setting operational objectives, it did not include meaningful guidance on how to reduce fundamental barriers to equitable palliative and end of life care – “it doesn’t really help me to address the barriers to equitable care that I encounter [as a palliative care specialist]” (Participant 653). Further, continuing difficulties in coordinating care, particularly in the context of under-resourcing and difficulty sharing records, were noted.

Finally, although the self-assessment tool was upheld as extremely useful in gap analyses, identifying how services should be developed, and other fundamental issues in practically addressing the identified gaps, were highlighted. Several participants described the difficultly of securing stakeholder time to meaningfully commit to the process, and lack of control over resources in, for example, social care sectors, that can impact overall care provision. In major part, these issues related to securing the resources necessary to meaningfully develop or adapt services. As one participant summed it up: “The ambitions will only be achieved if system boards and networks recognise the gaps by reviewing current provision and work upon funding to close the gaps.” (Participant 737).

## Discussion

### Implications of findings for policy and practice

Although Ambitions related work was described for a range of care settings, examples undertaken in specialist palliative care and hospice settings predominated in the survey. Given the professional population likely to engage with the Framework, this focus is not unexpected. However, our findings are encouraging in that they suggest that the Framework is seen as relevant to service commissioning and development across palliative and end of life care. The findings indicate that there is the potential for uptake in other settings, including non-specialist and in the community, through partnership working and commissioning structures, especially under Integrated Care Systems; yet, more work is needed to enable this to occur. Participants noted in particular the need for resources and education to support service development and implementation. It is unclear from the evidence provided to what extent the Framework is able to raise standards in services, both within and beyond palliative care; there is scope for further quality improvement and evaluation work to answer such questions.

The majority of reported service examples focused on Ambition 1 (Each person is seen as an individual), followed by Ambition 3 (Maximising comfort and wellbeing). Both Ambitions reflect a wider, and longer-standing, discourse in palliative and end of life care about holistic, person-centred care [2, 3]. Since many of the services commenced prior to the publication of the Framework, it may be that, to some extent, the Framework is being used to legitimise ongoing work, providing a national agenda with which to do so. It may also indicate limited scope to develop new services. Further research would enhance understanding of relevant issues, such as, for example, resource availability, and the impact of the COVID-19 pandemic.

Ambition 6 (Each community is prepared to help) was the least likely to be cited. It was evident that respondents were not considering (in the survey) how they or their staff live and work in communities. Yet, if they did consider the communities they are already part of, we see this as a potential useful avenue due to their ability to provide formal and informal community leadership. The qualitative data indicates that respondents viewed it as strategically significant that community approaches were included in the Framework. However, some noted that there is a tension between community-based approaches, such as compassionate communities, and the overall NHS England approach to palliative and end of life care, which is more clinically focused. This is an area where further research is warranted – to both understand how Ambition 6 is being understood and enacted, as well as how it relates to NHS England’s and commissioners’ approaches to incorporating ‘community’ within palliative care.

Of the Framework foundations, ‘Education and training’ was by far the most frequently cited as necessary to develop and/or sustain the reported service. Survey qualitative data suggests a need for education and training to be ongoing, to both improve and maintain quality of care. Our findings suggest the value of considering the education and training needs of different groups of stakeholders across the entire spectrum of palliative and end of life care, and in relation to different provider groups, including those based in the community. Such consideration could encompass, for example, how needs may differ, how they might be addressed, and how stakeholders can be empowered to provide ongoing education and training, including making use of existing materials. Whilst ‘Education and training’ is foundational to all six Ambitions, it is particularly pertinent for Ambition 5 (All staff are prepared to care) and Ambition 6 (Each community is prepared to help), given the knowledge and skills required to meet these two Ambitions in practice.

The foundation ‘Involving, supporting and caring for those important to the dying person’ was also highly cited as important to service design and/or sustainability. However, service examples focused predominately on the dying person, with only a limited number directly relevant to carer and/or bereavement support. The importance of carers in the provision of end of life care [27, 28], and the impact of their experiences on longer-term health and social outcomes, including grief [29, 30] are, by now, well documented. The consequent benefit to be gained from an enhancement of this element of the Framework within a broader prioritisation of its underlying mission is therefore suggested.

A recurrent theme concerned the Framework’s provision of a shared language, and the contribution of this language to collaborative work across sectors and partners. That said, the term ‘ambitions’ was, on occasions, critiqued for its potential implication that work could strive for, but ultimately not realise, its end goal. Although infrequently expressed, this critique parallels other comments concerning the difficulty of operationalising the six Ambitions, and of determining progress against them. The Framework therefore appears to function primarily in terms of guiding principles and values for service development and appraisal, rather than providing explicit direction or benchmarks that might underpin such work. Given these issues, further development of the Framework that might bridge the gap between values and action is suggested, particularly around operationalisation of the Ambitions, and evaluation of the work in which they are involved. In this context, support on how to develop business cases for service development suggested by Ambitions related work (including that involving use of the self-assessment tool) would address some of the stated concerns. This support is especially important given that the majority of reported service examples were NHS and/or charitable funded. In both cases, budgets may be limited and/or unpredictable [31–33]. Other research also points to the importance of large-scale co-ordinated approaches for implementing end of life care policy [34].

The theme of co-design and co-production is strong within the Framework, with co-design featuring as a foundation for the Ambitions, and co-production being the acknowledged method through which the Framework was developed. By its very nature, co-design encompasses a broad and growing landscape of activity, with the term often used interchangeably with others such as ‘co-creation’ and ‘collaboration’ [35, 36]. Over 70% of respondents stated that patients/services users/clients and/or the public were involved in the design of the reported service example. Yet, just over half of respondents (53%) cited co-design as required to design or sustain their service; this was the least frequently cited foundation out of the eight listed in the Framework. It was also addressed infrequently in the qualitative data. Our findings suggest that the ethos of co-design and co-production has not necessarily carried over into service development and delivery, at least in terms of the examples captured by the survey. Given this diversity, and the potential confusion and lack of confidence these concepts may engender, it is important that the wide range of stakeholders potentially involved in palliative and end of life health and social care service provision need to know what they mean, how they can be enacted, and what they can achieve. There is, therefore, scope for policy and implementation work around ensuring relevant knowledge, understanding, and skill development in co-design.

Perceived limitations in awareness of the Framework suggest a need for improved availability of and access to the document, as well as enhanced referencing and explicit signposting to the Framework across relevant national and local policy-related documents. That participants were able to note a wide range of such documents and also difficulties in making connections, aligns with previous research evidence [18, 19] and further suggests a need for explicit linkages to be drawn within and across policy and practice guidance. The current issues around accessing the document (behind a login or found via several Partner websites) 5 may mean that it is less readily available to non-NHS organisations or potential new partners. Ready access is particularly important in the context of efforts to develop partnerships involving non-specialist and/or community organisations. There is scope for the development of supporting materials for a range of audiences, which describe what the Framework is, why it maters, and how it can be used. These materials could target non-specialist palliative and end of life care providers, as well as community organisations and wider public.

Our findings indicate considerable scope and appetite for enhanced sharing of practice and mutual learning. Approximately 20% of respondents reported using the Framework to draw on specific examples, and/or to identify partner organisations with an interest in/commitment to improving palliative and end of life care. These efforts included in respect of the listed Ambitions Partners. A desire for enhanced opportunities for knowledge exchange was evident from our qualitative data. Respondents were both willing to share their experiences and keen to learn from others. Since most examples came from hospice or specialist palliative care settings, there is potential benefit to be gained by fostering knowledge exchange across sectors and settings of palliative and end of life care, and for it (to be seen) to be led by non-specialists. These endeavours are particularly pertinent in the context of the ongoing development of Integrated Care Systems.

One overall issue with the Framework noted by respondents was a perceive inability for the Framework to address systemic issues, especially around the provision of equitable palliative and end of life care. The Ambition statements were agreed to reflect ‘good practice’ but the focus on localism and lack of additional resources, including continuing difficulties in coordinated care and shared records, meant that participants felt that there was a lack of meaningful guidance or support to make changes. Concerns about localism have also been raised by previous literature focusing on the Ambitions Framework [18, 19].

### Strengths and limitations

Our study delivered novel evidence concerning understanding and use of the Framework. As a survey, our ability to explore the multiple issues addressed was limited, although free text comments did enable valuable insight. Further exploration of these issues is warranted, to unpack the understandings and experiences captured here. Such in-depth evidence will be of particular value in driving future targeted development of the Framework and supporting resources and activity.

We made concerted efforts to reach a broad cross-section of relevant stakeholders and were successful in terms of the geographical reach and service setting. As the Ambitions Framework is targeted at the local level and across a wide range of stakeholders, there is no central register of relevant activity. We are therefore unable to comment on a ‘response rate’. The survey received 45 responses, which is more examples than previous research has captured. A longer survey window and/or different timings (e.g., not impacted by focus on COVID) may have further increased the number of responses received.

Whilst the Ambitions Framework is aimed at England, we received responses from other parts of the UK. We included these in our analysis as we were interested in mapping use of the Framework. Further research could examine the reasons for people/services adapting the Framework beyond England and how this may differ from adaptation within England.

Our reliance on respondent self-selection means that we cannot rule out the possibility of bias in findings. Greater involvement of respondents working outside non-specialist palliative and end of life care would have helped in this regard. However, there was a high degree of consistency in issues raised, and in understandings expressed in relation to these issues, increasing our confidence in the relevance of our findings in core respects.

## Conclusion

The survey generated valuable summary level evidence on uptake of the Framework across England. Important insights have been gained into current and past work, the factors impacting on this work, and the implications for future development of the Framework. Our findings suggest considerable positive potential of the framework to generate local action as intended. The findings also offer a valuable steer for research to further understand the issues raised, such as in-depth case study analysis. There is also scope for additional policy and implementation activity that might help to address these issues, including how to bridge the gap identifying service gaps and commissioning services to address the ambitions.

## Electronic supplementary material

Below is the link to the electronic supplementary material.

Supplementary Material 1

## Data Availability

A version of the data with free text responses excluded will be uploaded the Open University’s research repository, ORDO. It will be filed under the researchers’ existing ORDO project about the Ambitions Framework.

## Footnotes

1 These presentations are not publicly available; recordings of the webinar series were provided to the research team by a co-lead of the Ambitions Partners.

2 The survey was favourably reviewed by The Open University Human Research Ethics Committee (HREC/4162).

3 Distribution of the newsletter was delayed due to a focus on Covid-critical activities.

4 Please note that the number of characteristics varies between two and four due to the fact that some had joint positions.

5 After the survey closed, NHS England subsequently provided a website to host the framework documentation.
