# Supplementary material for: Ambitions for palliative and end of life care: mapping examples of use of the framework across England
Source: BMC Palliat Care. 2023 Jun 29;22:83. doi: 10.1186/s12904-023-01207-3 (PMC10308731; doi:10.1186/s12904-023-01207-3)
Supplement: Supplementary file 1 — Supplementary Material 1 [file 12904_2023_1207_MOESM1_ESM.pdf]

Ambitions Framework: Collecting Case Studies

Page 1: Introduction

Study Information

Welcome to the ‘Implementation of the Ambitions Framework’ survey. The ‘Ambitions for Palliative and End of Life Care: a national framework for local action’ was first launched in 2015 and relaunched in 2021 for another 5-year term. It outlines six ambitions seeking to improve care, support and experience of death, dying and bereavement. You can see a recent copy of the Ambitions document here.

We are conducting a survey of people involved in commissioning, designing and delivering palliative and end-of-life care services and related activities (broadly defined) to understand how the Framework is used. As your work involves such care, we are inviting you to participate. The survey is led by Dr Erica Borgstrom (The Open University), working with Ms. Claire Henry and Dr Joanne Jordan.

The survey is about how you and others have used the Framework when designing, developing and reviewing your services or range of activities. The survey will take around 15 minutes to complete. The Open University Human Research Ethics Committee (HREC reference number: XXXX) favourably reviewed the study. The survey is funded by NHS England and NHS Improvement.

As part of further research, we will select some of the services that are described in the survey as case studies for more in-depth investigation. It is possible that we may wish to contact you at a future date, to ask if you would consider taking part in this further research. If you are willing to be contacted, there is an option to include your email address at the end of the survey. We will not share your address with anyone outside of the research team. We will not identify you to anyone else, including others involved in

the work you describe.

Participation and Consent

Completing the survey is entirely voluntary. Your participation or non-participation will not be linked to, or impact upon, your work in any way. At the end of the survey, you have the option to enter a prize draw for a £25 gift voucher to either Amazon or Love2Shop.

Completing the survey confirms that you give consent for The Open University to use the data that you provide (i.e., your responses to the questions included in the survey).

After you have completed and returned the completed survey, you still have the right to withdraw from the study. You do not need to provide an explanation and there will be no consequences to yourself. If you want to withdraw, you can contact Erica Borgstrom (Erica.borgstrom@open.ac.uk). We will delete the information you have provided. Withdrawal is possible for one month after you have retuned the survey; after that time the information you have provided will have become aggregated into the collective findings as part of our process of analysis.

How do I take part?

We advise that you **complete the survey in one sitting** to ensure your answers are saved and submitted.

At the end, there is a 'Finish' button – once pressed, it will be delivered directly to the Open University research team. In the following weeks, you may receive a reminder email; if you have already returned a completed survey, please ignore it.

If you have any questions, please contact Erica Borgstrom (erica.borgstrom@open.ac.uk).

Data Protection, Confidentiality and Retention

The data you provide will be processed and stored in accordance with relevant Open University policies. This means that raw data will be seen only by the researchers undertaking this work. It will be stored on university encrypted or password-protected laptops and in a dedicated folder on the university secure server. It will not be shared with anyone outside of the research team university.

In compliance with the Open University’s Data Retention policy, anonymised data will be kept for a minimum of 10 years following project completion. After 10 years, all data will be destroyed.

Dissemination

We will summarise the findings of the study in a report for NHS England. This will include examples of services and practices. At the end of the survey, you have the option to include your email address to receive a copy of this report.

Findings may also be presented at practitioner and academic conferences and published in academic journals.

Following study completion, relevant research outputs will be available on Open Research Online (ORO), the OU research data repository, available at http://oro.open.ac.uk/.

Accessibility

The survey has been designed for use on a standard computer (desktop or laptop). Full accessibility on a handheld device (such as a smartphone or tablet computer) cannot be guaranteed. If you have a disability or an additional requirement that makes it difficult for you to complete the survey online, please email Erica Borgstrom (contact details provided above).

Thank you

Thank you for taking the time to read this introductory information. We hope that you have found it useful. Your contribution is very much appreciated.

When completing the survey, please click on the 'Next' button below to continue, and where available use the 'Previous' button to navigate back through the survey. Remember to click ‘Finish’ at the end.

Page 2: Information about your example

On this page, we will ask you several questions about the example you are telling us about. This could be a service, practice (either establishment or how you do your job) or kind of activity that you do or know about. Examples can range from formal NHS and Social Care services to informal community action. We use the term ‘service’ below to refer to this wide range of possible examples.

What is the title of the service? ✱ *Required*

What is the lead organisation involved?

Briefly describe the service, including its aims and the population(s) it serves: ✱ *Required*

What area(s) are covered by this service? Select 1 or more options from the list below. ✱ *Required*

Please select at least 1 answer(s).

- [ ] East of England

- London
- Midlands
- North East and Yorkshire
- North West
- South East
- South West
- Scotland
- Wales
- Northern Ireland
- Other

If you selected Other, please specify:

What setting(s) does the service cover? Select one or more of the options below. ✱
Required

Please select at least 1 answer(s).

- primary care
- secondary care
- social care
- care homes
- ambulance
- hospice
- community hospitals
- pharmacy
- voluntary
- housing
- specialist palliative care

- mental health
- prisons
- domicillary care
- education and training provider
- community organisation
- Other

If you selected Other, please specify:

When was the service established?

- Before 2015
- 2015
- 2016
- 2017
- 2018
- 2019
- 2020
- 2021
- Not Known
- Not yet established

Has the service been reviewed? This may be through, for example, audits, feedback surveys, formal evaluation, and/or research.

- Yes
- No

- Unsure
- Not applicable

Were patients/service users/clients and/or the public involved in the design of the service?

- Yes
- No
- Unsure
- Not applicable

How is the service funded? Select one or more options from the list below.

- [ ] NHS
- [ ] Local Government
- [ ] Charity donations
- [ ] Grant (e.g. local council)
- [ ] Research funding
- [ ] Private business
- [ ] Other

If you selected Other, please specify:

What other organisations are involved in this service?

Page 3: Ambitions Framework

In this section, we ask you questions about the Ambitions Framework in relation to the example you just provided. Here is a copy of the Ambitions document for your reference.

What primary Ambition from the Ambitions Framework best matches the service you have described? ✱ *Required*

- ○ Each person is seen as an individual (Ambition 1)
- ○ Each person gets fair access to care (Ambition 2)
- ○ Maximising comfort and wellbeing (Ambition 3)
- ○ Care is coordinated (Ambition 4)
- ○ All staff are prepared to care (Ambition 5)
- ○ Each community is prepared to help (Ambition 6)

What other Ambitions are applicable to your service? Select one or more from the list below.

- ☐ Each person is seen as an individual (Ambition 1)
- ☐ Each person gets fair access to care (Ambition 2)
- ☐ Maximising comfort and wellbeing (Ambition 3)
- ☐ Care is coordinated (Ambition 4)
- ☐ All staff are prepared to care (Ambition 5)
- ☐ Each community is prepared to help (Ambition 6)

From the list below, what factors were needed to design and/or sustain the service? Select one or more from the list. ✱ *Required*

- [ ] Personalised care planning
- [ ] Shared records
- [ ] Evidence and information
- [ ] Those important to the dying person
- [ ] Education and Training
- [ ] 24/7
- [ ] Co-design
- [ ] Leadership
- [ ] Other

If you selected Other, please specify:

How have you used the Ambitions Framework within your service? Select one or more from the options below. ✱ *Required*

- [ ] Guiding principles (values)
- [ ] Service Design
- [ ] Business Case (e.g. cite the document in a case)
- [ ] Cite in local policy or guidelines
- [ ] Used in commissioning
- [ ] Quality Improvement
- [ ] To identify partner organisations
- [ ] As a tool for reviews, evaluation, or audit
- [ ] Drawn on specific examples in the document
- [ ] Education and/or training
- [ ] Other

If you selected Other, please specify:

What challenges did you encounter when using the Ambitions Framework for your service/practice?

What has the Ambitions Framework enabled you to do that you may not have otherwise done?

How else do you think you or others could use the Ambitions Framework and its related documentation?

In this space, you can tell us any other thoughts you have about the Ambitions Framework.

If you used any other policy, guidance, or frameworks when designing or delivering your service, what are they?

Please use this space to tell us anything else you'd like to share about your examples and your thoughts about the survey.

You are almost done. At the bottom of the next page you can click 'Finish' to save and send your response.

Page 4: Submission Page & Optional Contact Details

Are you willing to be contacted by a member of the research team for a follow-up discussion about the service/practice? If yes, please enter your email address here. This will be stored with your survey responses.

Please enter a valid email address.

Would you like a copy of the final report? If yes, please enter your email address here. This is optional and will not be stored with your survey responses.

Please enter a valid email address.

If you'd like to be entered into our prize draw for a £25 voucher (for either Amazon or Love2Shop), please enter your email address here. This is optional and will not be stored with your survey responses.

Please enter a valid email address.

Page 5: Thank you

Thank you for taking part in this survey. Following study completion, relevant research outputs will be made available on Open Research Online (ORO), the OU research data repository, available at http://oro.open.ac.uk/.

If you have any questions about the study, please contact the principal investigator Dr Erica Borgstrom (erica.borgstrom@open.ac.uk).
